# Stigma Associated with Alcohol and Other Drug Use Among People from Migrant and Ethnic Minority Groups: Results from a Systematic Review of Qualitative Studies

**DOI:** 10.1007/s10903-023-01468-3

**Published:** 2023-03-28

**Authors:** Caitlin H. Douglass, Thin Mar Win, Stelliana Goutzamanis, Megan S. C. Lim, Karen Block, Gerald Onsando, Margaret Hellard, Peter Higgs, Charles Livingstone, Danielle Horyniak

**Affiliations:** 1 Burnet Institute Australia, 85 Commercial Road, Melbourne, VIC 3004 Australia; 2 Melbourne School of Population and Global Health, University of Melbourne, 207 Bouverie Street, Carlton, VIC 3053 Australia; 3 Burnet Institute Myanmar, 226 Wizaya Plaza, U Wisara Road, Yangon, Myanmar; 4 Monash School of Public Health and Preventive Medicine, Monash University, 553 St Kilda Road, Melbourne, VIC 3004 Australia; 5 Melbourne School of Social and Political Sciences, University of Melbourne, 420 John Medley Building, Parkville, VIC 3010 Australia; 6 Public Health Department, La Trobe University, Plenty Road, Bundoora, VIC 3086 Australia

**Keywords:** Stigma, Alcohol, Illicit drugs, Migrant

## Abstract

Stigma reduces access to alcohol and other drug (AOD) support. This systematic review explored perceptions and experiences of stigma associated with AOD use among migrant and ethnic minority groups. Qualitative studies published in English were identified using six databases. Two reviewers screened and critically appraised articles using the Joanna Briggs Institute Critical Appraisal Checklist for qualitative studies. Data were synthesised using best fit framework synthesis. Twenty-three studies were included. Stigma drivers and facilitators included stereotypes, socio-cultural norms, legal responses and precarious lived experiences. Stigma intersected with gender, citizenship, race and ethnicity and manifested though shame, exclusion, secondary stigma and discrimination in treatment. Outcomes and impacts included avoidance of services, emotional distress, isolation and loneliness. This review identified similar stigma experiences to other populations, however outcomes were complicated by precarious lived experiences and multiple stigmatised identities. Multi-level interventions are required to reduce AOD-related stigma for migrant and ethnic minority groups.

**Supplementary Information:**

The online version contains supplementary material available at 10.1007/s10903-023-01468-3.

## Introduction

Stigma is a complex process where people or groups are identified as different, less desirable or dangerous [1]. Stigmatised characteristics are labelled as socially important, associated with negative stereotypes and considered different from the norm [2, 3] which contributes to status loss, exclusion, unfair treatment and internalised shame [2] and negatively affects employment, housing, healthcare access, treatment compliance and existing medical conditions [4]. Stigma is also context dependent with characteristics considered ‘normal’ in some circumstances and discreditable in others [1, 5]. Importantly, stigma occurs within social, cultural, economic and political systems, where those in power create and maintain hierarchies and determine what is normal [6].

Alcohol and other drug (AOD) use and particularly, dependence are considered stigmatised characteristics through their invocation of otherness [5]. Evidence suggests people experiencing dependence are stigmatised in social circles, healthcare, media and legal systems [7]. People who inject drugs are stereotyped as immoral, irresponsible, deviant and dishonest [8] and people with alcohol dependence are blamed for consuming alcohol in socially unacceptable ways [9]. Subsequently, affected people are considered undeserving of empathy, trust or support [10]. Internalised stigma occurs when individuals apply negative stereotypes to themselves which may decrease self-worth [3, 11]. Anticipated stigma is the expectation of experiencing bias if a stigmatised condition is discovered [12]. People close to a stigmatised individual may also experience secondary stigma [12, 13]. AOD-related stigma contributes to limited treatment access, poor quality healthcare, and obstruction of evidence-based responses [8].

Evidence suggests the prevalence of AOD use is higher among the general population compared to migrant and ethnic minority groups (i.e. populations other than the dominant majority of a country based on numerical proportions and power positions) [14–17]. However, these groups may still use AOD and experience harms due to trauma, mental health conditions, and socio-economic inequalities [18]. Furthermore, migrant and ethnic minority groups face challenges in accessing AOD support, with stigma acting as a major barrier [19–21]. Although stigma is also a challenge for the general population, migrant and ethnic minority groups likely face additional barriers to accessing support including limited awareness of where and how to seek help, language barriers and few services that go beyond western concepts and are able to meet the holistic and complex needs of individuals [22–24]. Studies suggest culture, socio-economic status, race, and gender shape stigma attached to health conditions thus the experiences of people from migrant and ethnic minority backgrounds likely differs within and between groups [11, 13, 25]. AOD-related stigma may intersect with ethnicity and citizenship leading to ‘double stigma’ (i.e. being stigmatised for one’s background and AOD use) and increased discrimination (i.e. being unfairly or less favourably treated than others) [26, 27]. Secondary stigma may be salient for migrant and ethnic minority groups when expected to uphold their family’s reputation [12, 13].

### Rationale

Although stigma is commonly identified as a barrier to help-seeking among migrant and ethnic minority communities [28–30], few studies explore people’s experiences, the underlying drivers and powerful discourses and systems that enable stigma to unfold. Additionally, there is a lack of synthesised data on stigma and intersections with other characteristics [20, 23]. This study aims to systematically review and synthesise existing literature to understand perceptions and experiences of stigma associated with AOD use among people from migrant and ethnic minority backgrounds.

### Guiding Theory

The theory underpinning this work is described in the protocol [31]. Our review draws on the concepts of habitus, symbolic power and stigma power. ‘Habitus’ refers to people’s beliefs, attitudes, behaviours and knowledge which are shaped by experiences, positionality and social institutions [32]. Symbolic power is the ability to define what constitutes reality, and impose a legitimate version of the social world on others [33]. Stigma represents symbolic power because those who articulate orthodox discourses via the social order determine what is legitimate, valuable and worthy. Similarly, stigma power is a resource that perpetuates existing power arrangements, creates and maintains hierarchies and determines whether characteristics are valuable [6]. People with stigmatised characteristics are encouraged to ‘stay in’ to avoid negative cultural evaluation, ‘stay away’ from threatening environments and ‘stay down’ by accepting their lower worth [6].

Our review was further guided by the Health Stigma and Discrimination Framework which suggests multiple domains interact to produce stigma [12]. Drivers are negative factors that increase stigma (e.g. stereotypes and prejudice) and facilitators can increase or decrease stigma (e.g. norms, beliefs and policies). Drivers and facilitators determine whether someone is ‘marked’ with stigma which can intersect with other stigmatised characteristics including race, ethnicity, gender and class. Stigma can manifest as experiences (lived realities) and practices (beliefs, attitudes and actions towards stigmatised people) and lead to ‘outcomes’ for affected populations (e.g. help-seeking behaviours) and health and social ‘impacts’ (e.g. quality of life) [12].

### Objectives

This review aimed to synthesise and critically analyse qualitative evidence exploring stigma associated with AOD use among people from migrant and ethnic minority backgrounds. Review questions included:

1. What are the underlying drivers and facilitators of AOD-related stigma among migrant and ethnic minority groups?
2. How does stigma associated with AOD use intersect with other stigmatised characteristics among migrant and ethnic minority groups?
3. How does stigma associated with AOD use manifest as experiences and practices among people from migrant and ethnic minority backgrounds?
4. What are the outcomes and impacts of AOD-related stigma for people from migrant and ethnic minority backgrounds?

## Methods

Review methods are described in the protocol [31] in accordance with Preferred Reporting Items for Systematic Reviews and Meta-analysis (PRISMA) guidelines and Enhancing transparency in reporting the synthesis of qualitative research checklist [34, 35]. The broader review was designed to examine stigma associated with mental health and/or AOD use due to the high prevalence of mental health conditions among migrant populations and co-morbidity with AOD-related problems [36, 37]. This manuscript presents findings on AOD-related stigma. Given our review questions and objectives focused on understanding perceptions and experiences of stigma, a systematic review of qualitative evidence was deemed appropriate.

### Eligibility Criteria

We used the Sample, Phenomenon of Interest, Design, Evaluation, Research type (SPIDER) tool to construct inclusion criteria [38].

- *Sample* Studies must report results for participants from migrant or ethnic minority backgrounds including participants who report AOD use or disorders and their community members, caregivers or family.
- *Phenomenon of interest* Studies must explore stigma associated with alcohol or illicit drug use (including dependence). Stigma must be identified as an aim, research question, theme or major result.
- *Design* Qualitative methodologies and/or data collection techniques.
- *Evaluation* Stigma-related perceptions and experiences.
- *Research Type* Original peer-reviewed qualitative studies or other study designs with relevant qualitative components published in English from 1990 to November 2021.

We excluded:

- Quantitative studies.
- Media content, document or policy analyses.
- Grey literature.
- Abstracts, conference presentations, dissertations, systematic reviews, literature reviews and commentaries.
- Published in language(s) other than English.
- Studies with Indigenous or First Nation’s peoples who have unique experiences underpinned by colonisation, dispossession, and discrimination; we feel we cannot do justice to these populations within this review. Other studies have explored AOD-related stigma among Aboriginal communities [39, 40].
- Studies with migrants from main English-speaking countries who do not identify with an ethnic minority group and are less likely to experience power disparities.
- Studies with health professionals or service providers.
- Studies that do not explore stigma in-depth.
- Focused on tobacco or medicinal cannabis.
- Focused on prescription medication only; prescription medication has unique social and cultural circumstances (e.g. over prescribing and the role of pharmaceutical companies) that go beyond the scope of this review [41]. Few studies have explored stigma associated with non-medical use of prescription medication or related dependence in any population [41, 42]. Studies have been included in this review if they focus on drug dependence and mention that some participants were dependent on prescribed medications (e.g. prescribed opioids).

### Information Sources

We identified articles using MEDLINE, Embase, PsycINFO, CINAHL, Applied Social Sciences Index and Sociological Abstracts, searched to November 2nd 2021. We reviewed references of included studies and contacted stigma experts to identify additional sources.

### Search Strategy

We refined MeSH terms and key words with a librarian including (migrant and ethnic minority) AND (AOD use or mental health) AND (stigma) AND (qualitative research) (See Online Supplementary Material).

### Selection Process

**Fig. 1:**
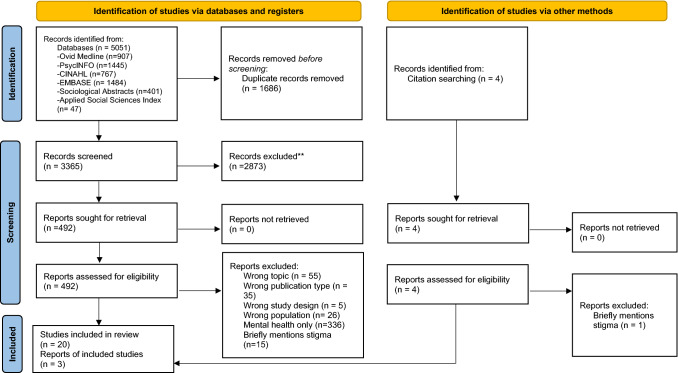
PRISMA flowchart of selection process. *Source* Page MJ, McKenzie JE, Bossuyt PM, Boutron I, Hoffmann TC, Mulrow CD, et al. The PRISMA 2020 statement: an updated guideline for reporting systematic reviews. BMJ 2021;372:n71. https://doi.org/10.1136/bmj.n71. For more information, visit: http://www.prisma-statement.org/

Three reviewers selected articles (CD, DH, TMW). CD downloaded citations from databases into Covidence (Covidence systematic review software, 2021, Veritas Health Innovation, Melbourne, Australia; www.covidence.org) and removed duplicates. Two reviewers screened titles, abstracts and full-text articles. Conflicts were managed through discussion and consensus. Figure 1 documents this process.

### Data Collection

CD extracted data from included studies in Covidence which was checked by TMW.

### Data Items

We extracted author, year of publication, country, city, participant characteristics, aim, methods and how stigma was captured (e.g. main focus, theme, sub-theme or described in the results but not exclusive focus).

### Critical Appraisal of Individual Studies

We critically appraised studies using the Joanna Briggs Institute (JBI) critical appraisal checklist for qualitative research [43, 44]. CD assessed every article and DH, TMW, KB, SG and PH assessed at least two articles each. Discrepancies were resolved through discussion, consensus and consulting a third reviewer.

### Synthesis Methods

Data were synthesised using best fit framework analysis. We coded data to the Health Stigma and Discrimination Framework [12, 45, 46] in Dedoose, a web application for managing, analysing, and presenting qualitative data (Dedoose Version 9.0.17, 2021. Los Angeles, CA: SocioCultural Research Consultants, LLC; www.dedoose.com). Two reviewers pilot tested the framework on five articles and adapted codes accordingly. One reviewer then applied the framework to each study’s results section including participant quotes and the author’s description of findings. Reviewers wrote memos, documented links between codes and refined interpretation through discussion. Data synthesis was shaped by the positionality of reviewers with backgrounds in AOD research, sociology, young people’s health, migrant inclusion and social cohesion.

### Confidence in Review Findings

We assessed level of confidence in review findings using the Grading of Recommendations Assessment, Development, and Evaluation—Confidence in the Evidence from Reviews of Qualitative research (GRADE-CERQual) [47]. We assessed methodological limitations of studies contributing to review findings, coherence of review findings, adequacy of data contributing to review findings and relevance of individual studies to review questions. Findings were graded as high, moderate, low or very low confidence.

## Results

### Study Selection

Our database search identified 5051 citations (Fig. 1). After removing duplicates, we screened 3365 titles and abstracts. Of these, 492 citations were eligible for full-text review and 20 met inclusion criteria. Three studies were added from searching reference lists, giving us a total of 23 included studies.

### Study Characteristics

Table 1 shows characteristics of included studies (n = 23). Studies generally included participants from a specific migrant and ethnic minority group (e.g. migrants from the former Soviet Union (FSU) in the US) [48, 49]. All studies were conducted in high-income countries except one in Iran (low-income) and one in China (upper-middle income) [50]. Most studies recruited participants undergoing treatment for substance use disorders [50–58] or reported illicit drug use [48, 49, 59, 60].

**Table 1:** Characteristics of included studies

Author	Aim	Migrant/ ethnic minority group and location	Methodology	Data collection	Participants	Location of stigma in results
Ayón and Carlson [51]	To explore cultural, familial, and contextual factors that impact Latina participants’ drug use and recovery process	Latina participants, US	Qualitative	Interviews	14 mothers who identified as Latina. Recruited from two drug rehabilitation centres. Mean age of 30 years. All born in the US	Sub-theme
Cottew and Oyefeso [52]	To examine why Bengali women who use substances do not access treatment	Bengali participants, England	Exploratory	Interviews	8 Bengali Muslim women who reported drug dependence recruited from two treatment settings. Mean age 23 years. All born in Britain	Described within results
Deilamizade et al. [50]	To explore the types of stigma and its consequences among Afghan refugees who use drugs	Afghan refugees, Iran	Qualitative	Interviews	27 male Afghan refugees who had completed detoxification from two residential drug treatment services. Mean age 26 years. 4 born in Iran	Main focus
Deng et al. [59]	To examine the extent and forms of stigma and resultant discrimination among people living with HIV who use drugs	Dai ethnic minority, China	Qualitative	Interviews, focus groups, observations and mapping	37 Dai community members including 9 people living with HIV who used drugs, 20 family members and 8 key informants recruited through community settings	Sub-theme
Guarino et al. [48]	To examine substance use patterns and social contextual factors among migrants from the FSU who use opioids	Migrants from the FSU, US	Exploratory	Interviews	10 participants who used opioids and identified as being Russian or of FSU descent recruited through services and community advertising. Mean age 31 years. Most lived in US for 10 + years	Sub-theme
Gunn and Guarino [49]	To explores youths’ perceptions of stigma attached to their opioid use within multiple life spheres	Migrants from the FSU, US	Qualitative	Interviews	26 young adults from FSU backgrounds with current opioid or heroin use or treatment for drug use disorders (mean age 26 years, mean age of immigration 7 years). 12 mothers from FSU who had children with opioid use disorders. Recruited through local services and community settings	Main focus
Gunn et al. [58]	To explore how mothers with intersecting experiences of incarceration and substance use navigate stigmas from family and romantic relationships	Black and Latina participants, US	Grounded theory	Interviews	19 Black and 4 Latina mothers completing their prison sentence in residential drug treatment. Age range: 19–56 years	Main focus
Higgs et al. [61]	To explore and document specific issues faced by females of Vietnamese ethnicity who use heroin	Vietnamese migrants, Australia	Exploratory	Interviews	24 female Vietnamese participants who used heroin recruited through street-based settings. Mean age 24 years	Theme
Ho and Maher [62]	To explore the influence of cultural beliefs and practices on vulnerability to blood-borne viruses among people from Vietnamese backgrounds who inject drugs and to identify barriers to accessing health and preventive programmes	Vietnamese migrants, Australia	Ethnography	Interviews and observations	58 Vietnamese participants who injected drugs recruited through street-based settings. Mean age 31 years. Mean duration of residence in Australia 18 years	Described within results
Horyniak et al. [60]	To examine exposure to, attitudes toward, and experiences of injecting drug use among marginalised African migrant and refugee youth from Melbourne’s western suburbs	African migrants, Australia	Grounded theory and field-based	Interviews	18 males born in any part of East Africa or Sudan who had ever used any illicit drugs recruited from field-based settings. Most reported regular alcohol and/or cannabis use. Age range: 19–36 years. Length of time residing in Australia: 6–14 years	Described within results
Jones et al. [53]	To explore the experience of Black women who had received substance use treatment, mental health services, or both	Black women, US	Exploratory	Focus groups	29 Black women who used mental health and/or substance use services. Mean age 37 years	Theme
Kour et al. [54]	To explore the treatment experiences of immigrant men living with co-occurring substance use disorders and mental health disorders	Migrants from a range of backgrounds, Norway	Exploratory	Interviews	10 males with immigrant backgrounds who experienced co-occurring substance use and mental health disorders and treated in Norway. Recruited through services and snowball sampling. Age range: 25–53 years. Migrated to Norway when they were 0–24 years, 2 born in Norway	Theme
Mallik et al. [63]	To explore imams’ perspectives and approaches toward Muslim Americans with substance use disorders	Muslim imams, US	Qualitative	Interviews	10 male imams recruited from mosques	Theme
Mantovani and Evans [55]	To understand factors contributing to drug using trajectories among British Bangladeshi men and women living in the East end of London	Bangladeshi, England	Qualitative	Interviews	15 Bangladeshi participants aged 18 years and over diagnosed with substance use disorder recruited from two drug treatment services. Mean age 32 years. 13 born in Britain	Theme
McCann et al. [21]	To identify help-seeking barriers and facilitators for anxiety, depression and alcohol and drug use problems in young people from recently established sub-Saharan African migrant communities	Sub-Saharan African migrants, Australia	Exploratory	Interviews and focus groups	28 African young people (aged 16–25 years, 89% lived in Australia for 6 years or less) and 41 African born parents and key community leaders recruited from community settings and African organisations	Theme
McCann et al. [64]	To explore the stigma experience surrounding mental illness and substance use, and its implications for improving help seeking, for youths and parents from sub-Saharan African immigrant communities	Sub-Saharan African migrants, Australia	Qualitative	Interviews and focus groups	28 African young people (aged 16–25 years, 89% lived in Australia for 6 years or less) and 41 African born parents and key community leaders recruited from community settings and African organisations	Main focus
Pinedo et al. [65]	To gain a better understanding of barriers to specialty substance use treatment among Latinos	Latinos, US	Qualitative	Interviews	20 Latino participants with recent substance use disorder recruited online. Mean age 39 years. 60% had used treatment in past 5 years. Most born in US	Sub-theme
Pinedo et al. [66]	To explore the barriers that may be contributing to Black-White disparities in the use of specialty alcohol and drug treatment	Black participants, US	Qualitative	Interviews	16 Black participants with recent substance use disorder recruited online. Mean age 42 years. 62% had used treatment in past 5 years	Sub-theme
Pinedo et al. [67]	To explore barriers to specialty substance use treatment programmes among women with recent substance use disorders by race/ethnicity	Black and Latino participants, US	Qualitative	Interviews	19 Black or Latina women with a recent substance use disorder recruited online. Black participants (mean age 28 years, 67% used treatment in past 5 years). Latina participants (mean age 40 years, 40% used treatment in past 5 years)	Sub-theme
Roy et al. [56]	To identify the factors that facilitate and hinder access to drug services in prison and to distinguish those that particularly affect Black and minority ethnic people in prison	Black and ethnic minority groups, England and Wales	Qualitative	Interviews and focus groups	111 Black and ethnic minority participants: 45 in prison receiving drug treatment, 31 in prison not receiving treatment, 8 who had previously been in prison and 27 community members	Theme
Scott and Wahl [57]	To examine the experience, manifestations, and impact of racial discrimination and substance use stigma	African Americans, US	Grounded theory	Interviews	10 African American males in recovery from substance dependence recruited through drug treatment facility. Mean age 34 years	Main focus
Suaalii-Sauni et al. [68]	To explore factors that support abstinence or responsible drinking amongst Pacific youth living in Auckland	Pacific Islanders, New Zealand	Qualitative	Focus groups	69 Pacific youth aged 15–25 years recruited from high schools or university. Mean age 18.5 years. 71% born in New Zealand	Described within results
Webber [69]	To examine the issues facing parents and siblings of people who use illicit drugs in the Vietnamese community	Vietnamese migrants, Australia	Exploratory	Focus groups	12 Vietnamese participants including 7 young people (aged 18–24 years) and 5 mothers with children aged 14–25 years	Described within results

*FSU* Former Soviet Union, *US* United States

### Critical Appraisal

Using the JBI Critical Appraisal Checklist, we rated included studies as low (n = 4), medium (n = 13) and high (n = 6) (Table 2). Most low-rated studies lacked information about research methodology.

**Table 2:** Critical appraisal of included studies

Author (Reference list no.)	Philosophical perspective and research methodology^a^	Research methodology and the research question ^b^	Research methodology and data collection methods^c^	Research methodology and data analysis^d^	Research methodology and interpretation^e^	Cultural or theoretical statement^f^	Influence of the researcher^g^	Participant voices^h^	Ethical research^i^	Conclusions^j^	Overall JBI rating
Ayón and Carlson [51]	Unclear	Unclear	Unclear	Unclear	Unclear	No	No	Unclear	Unclear	Yes	Low
Cottew and Oyefeso [52]	Yes	Yes	Yes	No	No	No	No	Unclear	Yes	Yes	Medium
Deilamizade et al. [50]	Unclear	Unclear	Unclear	Unclear	Unclear	No	No	Unclear	No	Yes	Low
Deng et al. [59]	Unclear	Unclear	Unclear	Unclear	Unclear	No	Unclear	Yes	Yes	Yes	Medium
Guarino et al. [48]	Yes	Yes	Yes	Yes	Yes	No	No	Yes	Yes	Yes	High
Gunn and Guarino [49]	Unclear	Unclear	Unclear	Unclear	Unclear	No	No	Yes	Yes	Yes	Medium
Gunn et al. [58]	Yes	Yes	Yes	Yes	Yes	Yes	Yes	Yes	Yes	Yes	High
Higgs et al. [61]	Unclear	Yes	Yes	Yes	Yes	No	No	Yes	Yes	Yes	High
Ho and Maher [62]	Unclear	Yes	Yes	Yes	Yes	Unclear	No	Yes	Yes	Yes	High
Horyniak et al. [60]	Unclear	Yes	Yes	Yes	Yes	Yes	Yes	Yes	Yes	Yes	High
Jones et al. [53]	Yes	Yes	Yes	Yes	No	No	No	Unclear	Yes	Yes	Medium
Kour et al. [54]	Unclear	Yes	Yes	Yes	Yes	Yes	Yes	Yes	Yes	Yes	High
Mallik et al. [63]	Unclear	Unclear	Unclear	Unclear	Unclear	Yes	Yes	Unclear	Yes	Yes	Medium
Mantovani and Evans [55]	Unclear	Unclear	Unclear	Unclear	Unclear	No	Yes	Yes	Yes	Yes	Medium
McCann et al. [21]	Unclear	Yes	Yes	Yes	Unclear	Unclear	No	Yes	Yes	Yes	Medium
McCann et al. [64]	Unclear	Yes	Yes	Yes	Yes	Yes	No	Yes	Yes	Yes	Medium
Pinedo et al. [65]	Unclear	Unclear	Unclear	Unclear	Unclear	Yes	No	Yes	Yes	Yes	Medium
Pinedo et al. [66]	Unclear	Unclear	Unclear	Unclear	Unclear	Yes	No	Yes	Yes	Yes	Medium
Pinedo et al. [67]	Unclear	Unclear	Unclear	Unclear	Unclear	Yes	Unclear	Yes	Yes	Yes	Medium
Roy et al. [56]	Unclear	Unclear	Unclear	Unclear	Unclear	No	No	Yes	Unclear	Yes	Low
Scott and Wahl [57]	Unclear	Yes	Yes	Yes	No	No	No	Unclear	Yes	Yes	Medium
Suaalii-Sauni et al. [68]	Unclear	Unclear	Unclear	Unclear	Unclear	No	No	Yes	Yes	Yes	Medium
Webber [69]	Unclear	Yes	Yes	Unclear	Yes	No	No	Unclear	Unclear	Yes	Low

Critical appraisal conducted using the JBI critical appraisal checklist for qualitative research

*JBI* Joanna Briggs Institute

^a^Is there congruity between the stated philosophical perspective and the research methodology?

^b^Is there congruity between the research methodology and the research question or objectives?

^c^Is there congruity between the research methodology and the methods used to collect data?

^d^Is there congruity between the research methodology and the representation and analysis of data?

^e^Is there congruity between the research methodology and the interpretation of results?

^f^Is there a statement locating the researcher culturally or theoretically?

^g^Is the influence of the researcher on the research, and vice- versa, addressed?

^h^Are participants, and their voices, adequately represented?

^i^Is the research ethical according to current criteria or, for recent studies, and is there evidence of ethical approval by an appropriate body?

^j^Do the conclusions drawn in the research report flow from the analysis, or interpretation, of the data?

### Results of Syntheses

**Fig. 2:**
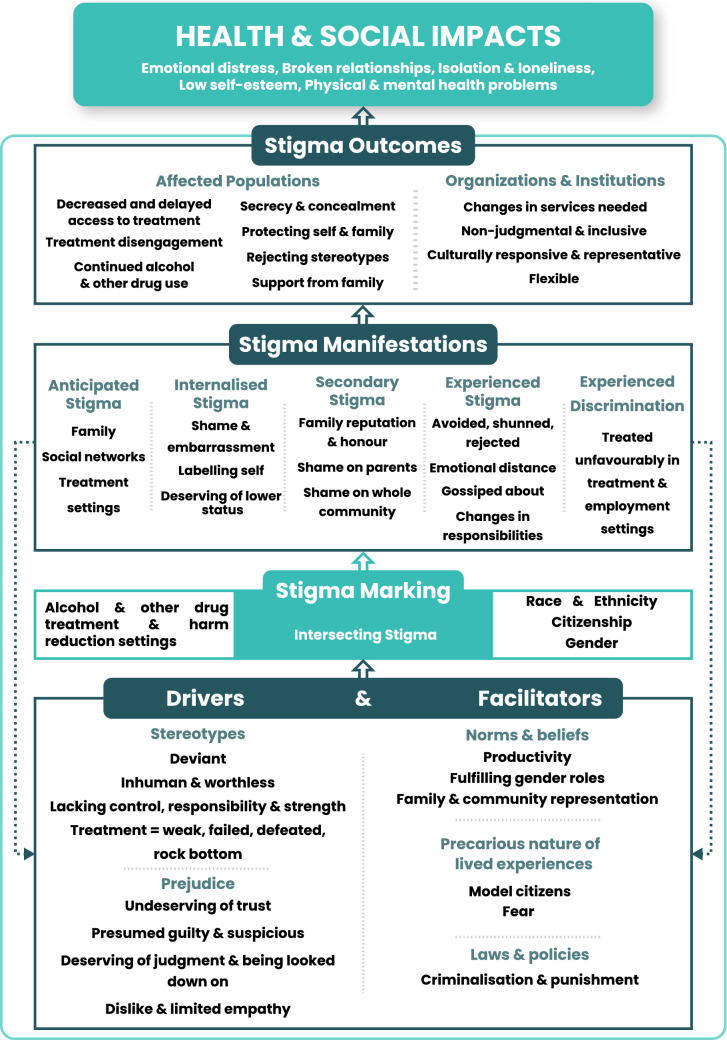
Modified Health Stigma and Discrimination Framework based on results from data synthesis

Most data from included studies corresponded with the Health Stigma and Discrimination Framework. After pilot testing, we added the code ‘Precarious nature of lived experiences’ under facilitators and combined ‘stigma experiences and practices’ given overlap in the data. Figure 2 shows a modified version of the framework.

Figure 2 was originally created by Stangl et al. [12] and has been reproduced under the terms of the Creative Commons Attribution 4.0 International License (https://creativecommons.org/licenses/by/4.0/) which permits unrestricted use, distribution, and reproduction in any medium with appropriate credit. Changes have been made within each domain to reflect the results from our data synthesis.

#### Stigma Drivers and Facilitators

Drivers and facilitators of stigma included underlying stereotypes, prejudice, norms and beliefs. Participants from migrant and ethnic minority backgrounds including family members, community leaders and those with first-hand experience of AOD treatment felt their communities perceived people who used illicit drugs as inferior, ‘garbage’, vectors for infectious diseases and ‘addicts’ unworthy of personhood [48, 49, 57, 59, 63].

“A drug user, this person is nothing, not human, they are dead already” (Migrant from FSU, mother whose son had an opioid dependence, US) [49, p. 9]

Participants believed stigma was driven by the perception that AOD use is a conscious choice [49, 51, 59, 60, 68] therefore individuals deserve judgment, punishment, blame, disrespect and distrust [49, 50, 52, 59]. Participants recruited from AOD treatment and community members believed negative attitudes were fuelled by limited knowledge of illicit drugs among families and communities [48, 51, 55, 64] Across different groups, normal and functional people were productive, educated, employed, and maintained family, household and financial responsibilities [48–50, 67, 68]. AOD use represented a violation of productivity norms and marked people as irresponsible others. Perceptions of AOD use were often hierarchical based on drug type and perceived impact on functionality [48, 49, 56, 67, 68]. Similar to existing literature [70, 71], the following participant distinguished themselves from other ‘dysfunctional addicts’ by engaging in downward comparisons:

“I would like you to understand that I am not a drug addict like others, that I don’t need it [drugs] every day to function. I have my serious side. I am a professional. When I go to work, I do my job well. I try to sleep 8 or 9 hours to be alert. This [drug use] is something I do when I do not have to go to work or anything” (Latina participant, alcohol and drug use disorder, US) [67, pp. 5–6]

Heroin and injecting drug use were considered especially discreditable whereas alcohol consumption was largely acceptable provided people could maintain responsibilities [48, 49, 65, 67]. These findings represent an order of symbolic power where people who consumed alcohol were considered more capable and valuable than people who used illicit drugs who were unquestionably hopeless and immoral [33].

“With alcoholics they live pretty functional lives in a family ...so drinking is thus accepted and not as stigmatised. They can work, maybe, buy something for the family. But a drug user, no money ...they can’t do anything well” (Migrant from FSU, mother whose son was experiencing drug dependence, US) [49, p. 9]

Studies described the precarious nature of lived experiences, particularly among refugees, including insecure employment, low wages, housing instability, social exclusion and the threat of repercussions for illegal activities [49, 50, 54, 60, 62, 64]. These precarious experiences increased pressure to demonstrate model citizenship, capitalise on opportunities, meet parental expectations and avoid deviant behaviours [48, 49, 68].

“I remember my mother [on] the plane… she told me, “This is your new beginning, new country, new people. Make the best of it” (Migrant from FSU experiencing drug dependence, US) [48, pp. 7–8]

Gender norms also facilitated AOD-related stigma. In six studies with diverse populations, women were expected to be primary caregivers, domesticated, strong and ‘sexually pure’ [49, 51, 55, 58, 59, 61]. Women who used drugs defied womanhood and were stereotyped as sexually deviant, unmarriable and unfit mothers, suggesting that AOD-related stigma upholds stereotypes of feminine virtue and reinforces traditional patriarchal roles [72].

“Boys can do anything they want . . . it doesn’t matter. If a girl does something then a guy won’t marry her because she’s been on the streets, she’s been on drugs so no one’s going to take her. She’s ruined”. (Muslim Bengali woman experiencing drug dependence, UK) [52, p. 182]

Religious norms and beliefs also facilitated AOD-related stigma [52, 59, 63]. Muslim imams described intoxication as haram (forbidden), sinful and a barrier to spiritual connection [63]. This finding was also echoed by Muslim Bengali women recruited from drug treatment settings who believed their heroin use defied religious and cultural norms [52]. These perceptions instituted a social reality where Muslims were considered legitimate if they attended mosque and performed good deeds but positioned as outsiders for AOD use. Stigma was also facilitated by social and cultural norms [48–50, 58–60, 62, 64, 65, 67–69]. In some communities, individual behaviour explicitly reflected upon family [62, 65, 68]. Young people from Pacific Islander backgrounds who consumed alcohol and Vietnamese people who injected drugs acknowledged the importance of carrying their family name and maintaining face to protect their families and communities from shame [62, 68]. This risk of damaging family and community reputations likely shaped preferences for solving problems within immediate families or trusted networks rather than professional services [21, 64, 67, 68].

Although evidence was limited, legal and policy responses facilitated stigma and created hesitancy to access support, particularly for opioid, heroin and injecting drug use [48, 49, 59, 62, 67]. Participants from an ethnic minority group in China, described harsh local drug strategies where people experiencing dependence were previously imprisoned, fined and denied rights to own property [59]. One Australian study described Vietnamese migrants residing in neighbourhoods with visible drug markets and heavy police presence, which created fear and unwillingness to approach harm reduction services [62].

#### Stigma Marking

Studies strongly suggested attending AOD treatment and harm reduction services posed a risk of being marked as problematic [48, 49, 52, 54–56, 62, 64–67]. This finding was particularly strong in studies that included people with first-hand experience of drug use or AOD-related disorders and less common in studies conducted with family and community members. Participants expressed concern about being identified as ‘addicts’ by members of their family, ethnic or local community.

“I wouldn’t want to go in person because what if I know somebody? Like, what if the people are my neighbours or what if their kids go to school with my kids? There is a huge negative stigma to people who have alcohol and drug problems […] I have heard people say like, you know, friends or at school or when I go on playdates, I hear people say like ‘Oh, that crack head’ or ‘that drug addict’ or ‘that tweaker’ and I am not trying to get called that. So, I wouldn’t go in person” (Latina participant experiencing AOD use disorder, US) [67, p. 7]

Participants who reported injecting drug use or experiencing an AOD use disorder were aware of their stigmatised identity and feared that accessing services legitimised treatment-related stereotypes. Participants perceived higher risk of marking where services were conspicuous or had long waiting times, for example at pharmacotherapy clinics. [62, 65, 66]

“You don’t want to wait outside the clinics because many other users are there. I just wanted to stop by, then go and get my dose quickly so no one can see me. But I often had to wait” (Vietnamese male who injected drugs, Australia) [62, p. 426]

The risk of stigmatisation within healthcare settings was further complicated by staff. Although some participants valued having service providers who shared their ethnicity, others feared confidentiality breaches [55, 62]. This participant perceived risk in visiting their doctor to be prescribed with methadone from a clinic with workers from the same community:

“Going there to the doctors to get my script … there are a lot of Bengali girls that work there, so, as soon as I walk in and there’s a surgery full of people: ‘Are you here for your script?’ It would be so loud that everyone would hear and they know the difference between a prescription and a script. And I would be like: ‘Oh, my God!’ trying to hide my face from them, thinking: ‘I hope they didn’t hear it’. Because they work in the surgery they know you’re on the script, so, they might know somebody that I know” (Migrant from Bangladesh with substance use disorder, England) [55, p. 129]

Some studies described intersectionality between AOD use stigma and other characteristics including citizenship status, race, ethnicity and gender. [49, 50, 52–54, 57, 59]. Males from Afghan refugee backgrounds treated for drug use disorders in Iran described being stereotyped as lazy and looked down upon by employers [50]. These refugee-related stereotypes combined with AOD-related stigma increased distrust and discrimination, highlighting their employer’s ability to exercise power through blame and exclusion.

“I was a tractor driver and worked for the Iranians. Until I was not addicted, there was no problem, but once I started taking drugs, the employer told me that you Afghans came to Iran and ruined our country, you do not work properly, you all are addicted. And eventually I argued with my employer, so he fired me and did not give me some of the money I demanded from him” (Afghan refugee who completed drug treatment, Iran) [50, p. 616]

Race and ethnicity were also important intersectional characteristics. In treatment settings, participants from African American, Caribbean, African and African Latino backgrounds reported experiencing 'double stigma' for their AOD use and for being Black, leading to unfair treatment, poorer health outcomes and difficulties obtaining employment [53, 57].

“Being a Black woman and an addict, being alienated and shamed not only because of my addiction, but based on my race and gender. Showing them my resume and having such a big hole in my work experience you know, and trying to figure out what lie I’m going to tell when they asked me what was you doing for ten years? What was you doing for ten years? So what’s my lie? I was raising my son. And what’s their view of me? Black uneducated, lazy, just making babies” (Black female participant who had received treatment for substance use, US) [53, p. 73]

This quote also demonstrates intersectionality between AOD use stigma and gender. In numerous studies, participants described how AOD use was perceived as worse for women than men [49, 52, 53, 59, 68], reflecting gender norms where women who used illicit drugs were considered irresponsible and unworthy of marriage.

#### Stigma Manifestations

Stigma manifestations included overlapping experiences and practices. Participants with AOD-related disorders, those who reported drug use and family members anticipated stigmatisation, which encouraged secrecy and prevented them from seeking support [49, 54, 59, 62, 64–67, 69]. Participants anticipated stigmatisation by families, friends and ethnic communities and feared being shunned, rejected, gossiped about and looked down upon [49, 58, 59, 62, 64–67, 69].

“I never contact others because I know they look down on me. After work, sometimes, I go to a public amusement room to watch TV but I do not dare to sit down, I just lean against the door or crouch near the gate. I also do not dare to visit my brother because I worry about gossip among his colleagues” (Male participant experiencing drug dependence from the Dai ethnic minority group, China) [59, p. 1568]

Participants experiencing AOD-related disorders also anticipated negative stereotyping from treatment and other healthcare professionals [62, 65, 67]. Black and Latina participants feared stigmatisation from White treatment providers who they felt lacked understanding of their experiences [67]. Studies documented first-hand experiences of stigma and discrimination within treatment settings [50, 53, 57, 62] particularly among participants with multiple stigmatised identities who felt treated poorly by service providers [50, 53, 57]. These findings emphasise treatment hierarchies where service providers decide who receives quality care.

“[Service providers] look at the Black people thinking we’re all addicts, or think that we’re mentally ill. . . you know when you come in all broken down, looking bad and the reception at the desk give you the look and turn her head on you just coming off the street and you’re looking for help, you know. But they don’t want to touch you or come near you” (Black female participant who received treatment for substance use disorder, US) [53, p. 72]

Studies conducted with people experiencing AOD-related disorders, participants who reported AOD use, family members and community members suggested discovery of AOD use or treatment access would cause secondary stigma for families [21, 49, 50, 52, 56, 57, 60, 62, 65, 68, 69]. Studies conducted with community members also identified that AOD use risked bringing shame on an individual’s ethnic or religious group, highlighting the need for secrecy [21, 59, 68]. Parents also feared being blamed for their child’s AOD use and perceived as failures by their communities [49, 69].

“They don’t want other people to look at them and go ‘shit, don’t hang around with that family because she has got a daughter on drugs’” (Vietnamese family member of a person who used illicit drugs, Australia) [69, p. 241]

Studies with participants with personal experience of drug use or AOD treatment provided further insight into stigma manifestations within relationships. Upon discovery of drug use, participants were avoided by friends and rejected from their families [49–51, 55, 59]. They described being stereotyped by their families as ‘junkies’, ‘weak’ and disappointments [49, 50, 58]. Some participants were separated from partners and children [50, 51], had responsibilities taken away and were excluded from family routines [50, 58, 59]. Family members may perceive this emotional and physical distance as a protective mechanism against secondary stigma.

“When my parents have meals with me, they often separate the tableware from mine or I just eat beside them. I know I am disgusting. They suspect I am infected with some diseases” (Male participant experiencing drug dependence from the Dai ethnic minority group, China) [59, pp. 1566–1567]

This participant’s description of themself as ‘disgusting’ is also imbued with internalised stigma and exemplifies the profound shame participants experienced. Studies conducted with individuals who had received AOD treatment and participants who reported injecting drug use provided additional insight into internalised stigma [51, 55–57, 61, 62]. Common labels participants assigned to themselves included ‘failures’, ‘junkies’ and ‘addicts’, reflecting stereotypes of worthlessness, weakness and deviance [49, 50, 55–57, 59, 60, 65]. The following quote emphasises how ongoing prejudice made participants feel deserving of their lower status:

“And people treated me like I was lower than them; like talking down to me. The sad part is that you kind of get used to people talking down to you like that…It made me feel like I was lower than people.” (African American male in recovery from substance dependence in the US) [57, p. 63]

Overall stigma manifested through personal experiences of stigma and discrimination, fear of experiencing anticipated and secondary stigma and internalisation of negative labels. Common practices included exclusion, gossip, stereotyping and prejudice.

#### Stigma Outcomes

Stigma manifestations encouraged secrecy and concealment of AOD use leading to negative outcomes for people from migrant and ethnic minority backgrounds. Participants hid their AOD use and service access from their families as a mechanism to protect themselves from judgment and rejection [48, 49, 52, 53, 60, 61, 64, 66, 67].

*“*No-one knows. If my parents find out then I am dead, they kill me. My addiction, no-one knows, so if I don’t use it from now on it’s even better for them [my parents], because then they don’t have to find out” (Vietnamese female who used heroin, Australia) [61, p. 686]

Community members and people with lived experience of drug use and treatment identified that people concealed their AOD use from their ethnic communities to protect their family’s reputation [54, 62–64]. Parents maintained secrecy by hiding AOD problems from their friends and relatives to uphold their family’s honour and avoid marginalisation, suggesting family members also experience negative outcomes including separation from social networks and decreased informal support [49, 55, 61, 64, 69].

“Of course, people who have never experienced this problem themselves will not understand it, that is why parents are in isolation. They can’t share this information, there is no one to listen to their pain...I had friends at work, women, whom I could not tell anything, my relatives whom I could not tell anything because they would not let me back into their house” (Migrant from FSU, mother whose son had an opioid dependence, US) [49, p. 12]

Stigma was also highly detrimental for accessing formal treatment and harm reduction services among people who used illicit drugs [49, 52, 54–56, 62, 65, 67]. Some people from migrant and ethnic minority backgrounds accessed treatment at late stages because they assumed it was reserved for ‘rock-bottom’, observable through homelessness, crime and failure to meet responsibilities [49, 55, 66, 67]. Results suggested stigma contributed to negative outcomes during and beyond treatment. Some participants who accessed support were negatively stereotyped and treated poorly by service providers leading to mistrust and early disengagement from treatment [54, 57]. After treatment, participants attempted to avoid AOD use however, often had limited employment opportunities and felt excluded by friends and family, which contributed to spending time with other people who used drugs [50, 59]. Continued AOD use became a mechanism for coping with difficult life circumstances leading to further stigma and discrimination [50, 59].

“I have been to DATC [drug and alcohol treatment centre] several times but I could not stop taking it. After I came back from DATC, I felt lonely, because no one really understood me except my ‘No. 4’ [heroin] friends, so I had to contact them and relapsed again” (Male participant experiencing drug dependence from the Dai ethnic minority group, China) [59, p. 1568]

A minority of studies described participants’ resilience and advocacy [51, 53, 54, 58]. Participants recruited from AOD treatment settings rejected negative stereotypes and advocated to reduce stigma rather than demonise individuals [51, 53, 54]. Black and Latina women in residential treatment challenged the label of unfit mothers [58]. Additionally, some Latino participants reported their families supported them to seek help, leading to positive treatment experiences [51].

“They supported me. When they knew I had a problem they all got together and they let me know that they were there for me. They’re willing to be there to help me do whatever it takes to recover” (Latino participant in substance use treatment, US) [51, p. 68]

Although evidence was limited, participants believed services should be non-judgmental, welcoming and inclusive by increasing cultural responsiveness and representation [53, 63, 67]. Some participants perceived places of worship as inclusive and supportive in AOD treatment [57, 63, 66]. Black and Latino participants believed treatment programmes would benefit from employing staff from a similar culture and gender to patients [67]. However, this preference may differ for participants who feared for their confidentiality [55, 62]. Participants believed services could use less overt and stigmatising labels for AOD programmes and incorporate AOD-related information into general health and wellbeing programmes [21, 54].

#### Stigma Impacts

Few studies explored the long-term health and social impacts of stigma among people from migrant and ethnic minority groups. However, some studies conducted with people with lived experience of drug use or AOD-related disorders suggested stigma caused emotional distress, relationship breakdowns, isolation and loneliness [49, 50, 57, 59]. Stigmatisation also negatively impacted psychological wellbeing particularly where participants internalised stigma and experienced low self-esteem [50, 59]. These findings suggest people from migrant and ethnic minority backgrounds who experience stigma likely have reduced quality of life.

“I feel bad about myself; I feel like I am miserable; I’m alone; I don’t like myself.” (Afghan refugee who completed drug treatment, Iran) [50 p. 618]

Additionally, studies suggested personal experiences and fear of stigma led to participants and their family members hiding AOD use and avoiding support services. Given potential treatment benefits, delayed or no access to support likely has negative implications for physical and mental health.

### Level of Confidence in Review Findings

Table 3 presents a summary of review findings and level of confidence. Our confidence ranged from very low (i.e. findings supported by few studies in limited settings with methodological limitations) to high (findings supported by multiple studies with rich data).

**Table 3:** Summary of review findings and level of confidence

Summary of review finding	Studies contributing to finding (Reference list number)	Assessment of methodological limitations	Assessment of relevance	Assessment of coherence	Assessment of adequacy	GRADE-CERQual assessment of confidence in the findings
*Drivers and facilitators*						
1. Underlying stereotypes framed people from migrant and ethnic minority backgrounds who used illicit drugs and/or experienced dependence or substance use disorders as immoral, deviant, inhuman, worthless and out of control	[48, 49, 51–53, 56, 57, 59–61, 63, 67, 69]	Minor concerns	Minor concerns	No or very minor concerns	No or very minor concerns	High confidence
2. Productivity norms, family responsibilities and precarious lived experiences underpinned the stereotypes of people who consumed alcohol, used illicit drugs or needed treatment for AOD related problems	[48–51, 60, 62, 64, 65, 67, 68]	Minor concerns	Minor concerns	Minor concerns	Moderate concerns	Moderate confidence
3. Women from migrant and ethnic minority backgrounds faced additional stereotypes for AOD use related to sexual deviance, caregiving, domesticity and marriage	[49, 51, 52, 58, 59, 68]	Moderate concerns	Moderate concerns	No or very minor concerns	Moderate concerns	Low confidence
*Stigma marking*						
4. Treatment and harm reduction settings were a risk environment for stigmatisation	[48, 49, 52, 54–56, 62, 64–67]	Minor concerns	Minor concerns	No or very minor concerns	No or very minor concerns	High confidence
5. Stigma associated with AOD use intersected with stigma towards citizenship status, race, ethnicity and gender	[49, 50, 52–54, 57–59]	Minor concerns	No or very minor concerns about relevance	Moderate concerns	Moderate concerns	Moderate confidence
*Stigma manifestations*						
6. People from migrant and ethnic minority backgrounds experienced stigma through rejection, avoidance and exclusion by family and friends	[49–51, 55, 57, 59, 66]	Moderate concerns	No or very minor concerns	No or very minor concerns	Minor concerns	Moderate confidence
7. People from migrant and ethnic minority backgrounds internalised negative labels and experienced shame for their AOD use and related disorders	[49–51, 55–57, 59–62, 64, 65, 69]	Minor concerns	No or very minor concerns	Minor concerns	Minor concerns	High confidence
8. AOD use was considered shameful for the families of people from migrant and ethnic minority backgrounds	[21, 49, 50, 52, 54, 56, 57, 60, 62, 65, 68, 69]	Minor concerns	No or very minor concerns	No or very minor concerns	Minor concerns	High confidence
*Stigma outcomes*						
9. People from migrant and ethnic minority backgrounds concealed their AOD use and avoided seeking help to protect themselves and their families from stigma	[48, 49, 51, 52, 54–56, 60–67, 69]	Minor concerns	No or very minor concerns	No or very minor concerns	No or very minor concerns	High confidence
*Health and social impacts*						
10. Stigma had negative impacts on relationships, wellbeing and self-esteem	[49, 50, 57, 66]	Moderate concern	Moderate concerns	No or very minor concerns	Serious concerns	Very low confidence

*AOD* Alcohol and other drug, *GRADE-CERQUAL* grading of recommendations assessment, development, and evaluation-confidence in the evidence from reviews of qualitative research

## Discussion

This systematic review explored AOD-related stigma among migrant and ethnic minority communities. Family and community members were aware of the negative stereotypes driving AOD-related stigma and the risk of secondary stigma for families. Studies conducted with participants who reported drug use or an AOD-related disorder provided additional insight into the intersectional nature of stigma, services as a risk environment for stigma marking and discrimination, internalised stigma, the importance of protecting family and attempts to challenge stigma.

Our findings parallel with studies among non-migrant and ethnic minority groups, suggesting AOD-related stigma transcends populations and settings. People who use AOD, particularly illicit drugs like heroin are commonly stereotyped as ‘addicts’ unable to contribute meaningfully to society [5, 73]. Similar to other evidence, illegal drugs, particularly injecting drug use were more stigmatised than alcohol consumption, suggesting a drug’s legal status is a major facilitator of stigma [74, 75]. Across different populations, people with substance use disorders were perceived as weak and deserving of lower status [5, 74, 76]. These stereotypes distinguish ‘normal’ and ‘responsible’ people (i.e. those who are not dependent) and ‘deviant’ people who fail to uphold good morals and therefore deserve social devaluation [2]. People from non-migrant and ethnic minority backgrounds have reported similar stigma manifestations, including negative AOD treatment experiences [5, 74, 76].

Despite similarities, our findings suggest people from migrant and ethnic minority backgrounds are increasingly vulnerable to negative stigma outcomes due to precarious lived experiences and the intersection of stigmatised characteristics. Limited employment opportunities, low wages, housing instability and social exclusion create climates where people from migrant and ethnic minority backgrounds feel compelled to be productive [77]. Other studies suggest precarious experiences cause stress and anxiety and create difficulties accessing healthcare services [78, 79]. Our findings also suggested AOD-related stigma intersected with citizenship status, race, ethnicity, and gender, which potentially worsened outcomes for individuals with multiple stigmatised identities. These intersectional stigmas positioned affected individuals at the bottom of social hierarchies, encouraging them to avoid situations where they may be identified as problematic [6]. Similar observations have been identified among Aboriginal Australians living with hepatitis C, who described overlapping stigma related to hepatitis C, injecting drug use and their Aboriginal identity [80].

Review findings indicated people’s families and communities may also be negatively labelled for an individual’s AOD use. This finding reflects broader literature on racism and representation where people from migrant and ethnic minority communities are pressured to positively represent their community or risk all members being negatively tainted [81, 82]. Subsequently, people from migrant and ethnic minority backgrounds stayed away from services to protect themselves, their families and communities from negative stereotyping [6]. This experience is likely isolating and distressing for people who rely on families and communities for support.

### Implications

Review findings highlight multi-faceted interventions are required to reduce the negative outcomes and impacts of stigma associated with AOD use among migrant and ethnic minority groups. Actions are needed to address internalised stigma, negative manifestations and the political and power structures that allow stigma to unfold. Another systematic review investigated interventions to reduce stigma associated with substance use disorders [83]. Results suggested therapeutic interventions may reduce internalised stigma and motivational interviewing and sharing positive stories about people with substance use disorders reduced stigmatising attitudes among the general public. However, the body of evidence was small and did not target migrant and ethnic minority groups. To develop interventions, services need to work in partnership with migrant and ethnic minority groups to ensure programme messages, format and delivery are relevant and appropriate [84]. In Australia, AOD services, community groups and research institutes have collaborated to target AOD use and stigma among migrant and ethnic minority groups, including partnerships with South Sudanese, Chin (an ethnic minority group from Myanmar) and Indian communities [85, 86]. Although these culturally targeted approaches may be useful for reducing internalised and secondary stigma, it is likely other approaches are needed to address stigma within treatment settings.

A systematic review reported education programmes targeting medical students and professionals improved attitudes towards people experiencing substance use disorders [83]. Similar findings are evident in the HIV literature; studies suggest providing skills-based training for hospital staff and delivering brief electronic interventions targeting the drivers of stigma reduced service provider’s prejudice and intentions to stigmatise people living with HIV [87–89]. However, education alone is unlikely to achieve large reductions in stigma [90]. Within treatment settings, policies and practices must promote inclusion for people from migrant and ethnic minority groups who may experience intersectional stigma. More broadly, our findings indicated that stigma was facilitated by a drug’s legal status, suggesting decriminalisation may reduce stigma towards illegal drugs. This approach is supported by evidence from Portugal where decriminalisation led to reductions in drug-related harms and increased access to treatment [91].

### Future Research

Our review highlights opportunities for future research. Most studies were conducted in high-income countries, likely because our review only included peer-reviewed studies published in English [92]. Future reviews could focus on studies in low-income countries and published in languages other than English. We excluded papers focused on prescription medication only given the unique social and cultural circumstances that go beyond the scope of this review [41]. Some studies suggest there may be lower levels of stigma associated with prescription medication and related dependence compared to other substance use disorders [41, 42]. Studies have documented non-medical use of prescription medication among migrant populations however, most do not focus on stigma suggesting further research in this area is warranted [41, 93–95]. No studies specifically explored stigma associated with AOD use among people seeking asylum, a group who may experience trauma, long periods of uncertainty and significant mental health challenges [96]. Most studies recruited participants who were born in the country where the study was conducted or had lived there for over a decade, suggesting further research is needed with newly-arrived migrants and refugees. Most studies also recruited participants from treatment settings or reported that most participants had previously accessed treatment. Experiences of stigma may differ among those who have not accessed treatment before.

In our review, we had low confidence in the impacts of stigma because few studies explored this domain. Longitudinal studies examining stigma impacts are required to inform interventions. We were moderately confident AOD-related stigma intersected with citizenship status, race, ethnicity and gender. However, prior to analysis, we believed age, class, other health conditions, and sexual identity may also be important thus further research into intersectional factors is needed [96–98]. A minority of studies explored stigma resistance and challenging stigma. Future studies should explore these topics to uncover stories of hope and resilience and capitalise on existing stigma management strategies within communities and activist groups. Finally, future research should adapt existing stigma-based interventions and increase their relevance for migrant and ethnic minority groups by accounting for intersectional stigma, interpersonal relationships and precarious lived experiences. Interventions should be developed and evaluated in partnership with services and communities to determine their acceptability, feasibility and effectiveness [99].

### Limitations in the Body of Evidence

Studies in this review were limited by insufficient detail on methodology, with few studies discussing philosophical perspectives, positionality or theory. Participatory studies that engage people from migrant and ethnic minority backgrounds in research development are required to ensure suitable methods are used [100]. Researchers should consider how their positionality including ethnicity, cultural background and relationship with participants shapes the study conduct and results [101]. Studies could also be strengthened by using clear definitions of stigma and related concepts, given many studies use terms interchangeably [10].

### Strengths and Limitations of This Review

This review makes a valuable contribution to the evidence by synthesising studies on AOD-related stigma among migrant and ethnic minority groups. Findings should be interpreted with limitations in mind. Our search included peer-reviewed manuscripts published in English thus we likely missed findings from grey literature and studies written in other languages. Due to time and resource constraints, multiple reviewers assessed the quality of articles, which may have increased inconsistencies however, all reviewers received detailed instructions. Finally, qualitative systematic reviews involve taking results from their original context and addressing new questions, which is complex with studies across multiple countries and cultures [92]. To retain some context, we coded the original author’s interpretation of results during analysis.

## Conclusion

Our results suggest migrant and ethnic minority groups report similar underlying drivers, facilitators, markers and manifestations of stigma compared to mainstream populations. However, outcomes of stigma are complicated for migrant and ethnic minority groups by precarious lived experiences and the convergence of multiple stigmatised characteristics. Multi-faceted interventions developed in partnership with migrant and ethnic minority communities are required to reduce the occurrence and negative impacts of AOD-related stigma.

### Supplementary Information

Below is the link to the electronic supplementary material.

Supplementary file1 (PDF 342 kb)

## Declarations

### Registration and Protocol

This review is registered with PROSPERO (ID CRD42021204057). The protocol is available online [28].
