# Supplementary material for: Stigma Associated with Alcohol and Other Drug Use Among People from Migrant and Ethnic Minority Groups: Results from a Systematic Review of Qualitative Studies
Source: J Immigr Minor Health. 2023 Mar 28;25(6):1402–25. doi: 10.1007/s10903-023-01468-3 (PMC10632266; doi:10.1007/s10903-023-01468-3)
Supplement: Supplementary file 1 — Supplementary file1 (PDF 342 kb) [file 10903_2023_1468_MOESM1_ESM.pdf]

Online Supplementary Material: Search Strategy

Ovid Medline, PsycINFO and Embase

#	Searches
1	african continental ancestry group/
2	asian continental ancestry group/
3	ethnic groups/ or african americans/ or arabs/ or asian americans/ or hispanic americans/
4	acculturation/
5	"emigrants and immigrants"/
6	Minority Groups/
7	"Transients and Migrants"/
8	Refugees/
9	Refugee Camps/
10	cultural diversity/
11	Human Migration/
12	undocumented immigrants/
13	CALD.mp.
14	(transient* or migrant* or immigrant* or emigrant* or refugee* or migration).mp.
15	(asylum adj2 seek*).mp.
16	((ethnic* or racial) adj (minor* or group* or population* or communit* or origin* or people* or background*)).mp.
17	(cultur* adj3 divers*).mp.
18	(displac* adj1 (internal* or forced or mass or person* or people* or population*)).mp.
19	or/1-18
20	Mental Health/
21	Mental Disorders/
22	Mentally Ill Persons/
23	Anxiety/
24	anxiety disorders/ or agoraphobia/ or anxiety, separation/ or neurotic disorders/ or obsessive-compulsive disorder/ or hoarding disorder/ or panic disorder/ or phobic disorders/ or phobia, social/
25	depression/ or mood disorders/ or depressive disorder/ or depression, postpartum/ or depressive disorder, major/ or depressive disorder, treatment- resistant/ or dysthymic

disorder/ or premenstrual dysphoric disorder/ or seasonal affective disorder/ or cyclothymic disorder/

26 stress disorders, traumatic/ or psychological trauma/ or historical trauma/ or stress disorders, post-traumatic/ or stress disorders, traumatic, acute/

27 self-injurious behavior/ or self mutilation/ or suicide/

28 schizophrenia/ or schizophrenia.mp.

29 Psychology, Clinical/ or stress, psychological/

30 mental health services/ or community mental health services/ or counseling/ or social work, psychiatric/

31 mental health recovery/ or psychiatric rehabilitation/

32 (mental* adj1 (health* or ill* or well* or disease* or disorder* or problem* or condition* or issue*)).mp.

33 (depress* or anxiet* or trauma*).mp.

34 Psychotic Disorders/ or psychotic.mp. or psychosis.mp.

35 (PTSD or posttrauma* or post?trauma* or post trauma*).mp.

36 (affective disorder? or dysthymi* or agoraphobi* or panic).mp.

37 "Diagnosis, Dual (Psychiatry)"/ or dual diagnos*.mp.

38 or/20-37

39 substance-related disorders/ or alcohol-related disorders/ or alcohol-induced disorders/ or psychoses, alcoholic/ or alcoholic intoxication/ or alcoholism/ or amphetamine-related disorders/ or cocaine-related disorders/ or drug overdose/ or inhalant abuse/ or marijuana abuse/ or narcotic-related disorders/ or opioid-related disorders/ or heroin dependence/ or morphine dependence/ or opium dependence/ or phencyclidine abuse/ or psychoses, substance- induced/ or substance abuse, intravenous/ or substance abuse, oral/

40 drinking behavior/ or alcohol abstinence/ or alcohol drinking/ or binge drinking/ or alcohol drinking in college/ or underage drinking/

41 Drug-Seeking Behavior/

42 Drug Users/

43 illicit drugs/

44 Substance Abuse Treatment Centers/

45 Alcoholics/

46 crack cocaine/

47 amphetamines/ or methamphetamine/

48 (PWID or PWUD).mp.

49 (cannabis or marijuana).mp.

50 (heroin or opiate* or opium or opioid* or narcotic* or cocaine).mp.

51 AOD.mp.

52 (drug adj2 (illicit* or illegal* or inject*)).mp.

53 ((drug* or substance* or alcohol*) adj2 (addict* or dependen* or problem* or abuse* or treat*)).mp.

54 alcoholic beverages/ or absinthe/ or beer/ or wine/

55 (alcohol* adj (drink* or consum*)).mp.

56 (khat or qat).mp.

57 (amphetamine* or methamphetamine*).mp.

58 (ecstasy or mdma or ketamine or hallucinogen* or inhalant*).mp.

59 or/39-58

60 38 or 59

61 dehumanization/ or prejudice/ or social discrimination/ or social distance/ or social marginalization/ or social stigma/ or stereotyping/ or stereotyped behavior/

62 social values/

63 Taboo/

64 rejection, psychology/ or scapegoating/

65 stigma*.mp.

66 stereotyp*.mp.

67 prejudic*.mp.

68 (social* distanc* or ostraci?*).mp.

69 (dehumanis* or dehumaniz*).mp.

70 (dishonor* or dishonour*).mp.

71 ((social* or communit* or cultural* or famil* or ethnic* or racial*) adj (exclusion* or exclud*)).mp.

72 (marginalis* or marginaliz*).mp.

73 taboo*.mp.

74 shame*.mp.

75 (discriminat* adj2 social*).mp.

76 or/61-75

77 community-based participatory research/ or grounded theory/ or qualitative research/

78 interview/

79 Interview, Psychological/

80 focus groups/ or interviews as topic/

81 anthropology/ or anthropology, cultural/

82 qualitative*.mp.

83 (phenomenolog* or phenomenograph*).mp.

84 (ethnograph* or ground* theor*).mp.

85 interview*.mp.

86 (focus group* or group* discuss*).mp.

87 lived experience*.mp.

88 (thematic* or theme$).mp.

89 ((participatory adj2 (study or research or project)) or photovoice* or photo?voice).mp.

90 narration/ or narration.mp.

91 mix* method*.mp.

92 or/77-91

93 19 and 60 and 76 and 92

94 limit 93 to english language

95 limit 94 to yr="1990 -Current"

Sociological Abstracts and Applied Social Sciences Index and Abstracts

noft(migrant* OR emigrant* OR refugee* OR asylum seek* OR people* seek* asylum* OR ethnic minor* OR ethnic group* OR acculturation OR immigrant* OR cultural* divers* OR cultural* AND linguistic* divers* OR CALD OR racial minor* OR forc* displac* OR non?english speak* OR NESB OR english as second language OR english second language) AND noft(mental* health OR mental* ill* OR mental* well* OR mental* disorder* OR mental* condition* OR depress* OR anxiet* OR post traumatic stress OR posttraumatic stress OR post?traumatic stress OR PTSD OR trauma* OR psychosis OR schizophreni* OR mood disorder* OR suicid* OR substance abuse* OR drug abuse* OR drug use* OR drug usage OR drug overdose* OR substance abuse* OR drug addict* OR drug dependenc* OR inject* drug* OR PWID OR PWUD OR illicit drug* OR illegal drug* OR substance disorder* OR dual diagnos* OR drug seek* OR cannabis OR marijuana OR heroin OR cocaine OR ecstasy OR mdma OR narcotic* OR ketamine OR hallucinogen* OR inhalant* OR amphetamine* OR methamphetamine* OR khat OR qat OR drinking OR alcoholism OR alcoholic* OR alcohol* drink* OR alcohol consum* OR alcohol usage OR alcohol use* OR alcohol abuse* OR alcohol addict* OR alcohol dependen*) AND noft(stigma* OR discrimination OR prejudice* OR stereotyp* OR dehumani?* OR social marginali?ation OR taboo* OR shame* OR rejection) AND noft(qualitative* OR grounded theor* OR participatory research OR focus group* OR interview* OR grop discuss* OR phenomenolog* OR ethnograph* OR thematic* OR theme* OR photo voice OR photovoice OR photo-voice OR mix* meth* OR lived experience* OR narration*) AND (bdl(10000263 1007480) AND stype.exact("Scholarly Journals") AND la.exact("ENG") AND pd(19900101-20211231))

CINAHL

#	Query
S88	S87 - Limiters - Publication Year: 1990-; English Language<br>Search modes - Boolean/Phrase
S87	S15 AND S56 AND S70 AND S86
S86	S71 OR S72 OR S73 OR S74 OR S75 OR S76 OR S77 OR S78 OR S79 OR S80 OR S81 OR S82 OR S83 OR S84 OR S85
S85	mix* method*
S84	narration OR narrative*
S83	(participatory N1 (study or research or project)) or photovoice* or photo? voice)
S82	thematic* or theme$
S81	lived experience*
S80	focus group* or group* discuss*
S79	interview*
S78	phenomenolog* or phenomenograph*
S77	qualitative*
S76	SU Focus Groups
S75	MH Narratives
S74	MH Interviews
S73	SU Thematic Analysis or SU Phenomenological<br>Research or SU Grounded Theory or SU Ethnological Research or SU Ethnographic Research or SU Action Research
S72	MH Qualitative Validity
S71	MH Qualitative Studies
S70	S57 OR S58 OR S59 OR S60 OR S61 OR S62 OR S63 OR S64 OR S65 OR S66 OR S67 OR S68 OR S69
S69	discriminat* N1 social*
S68	taboo*
S67	marginalis* or marginaliz*

S66	prejudic*
S65	shame*
S64	dehumanis* or dehumaniz*
S63	ostraci?*
S62	stigma* or stereotyp*
S61	SU Stigma
S60	MH Discrimination
S59	SU Dehumanization
S58	SU Stereotyping
S57	MH Prejudice
S56	S33 or S55
S55	S34 or S35 or S36 or S37 or S38 or S39 or S40 or S41 or S42 or S43 or S44 or S45 or S46 or S47 or S48 or S49 or S50 or S51 or S52 or S53 or S54
S54	ecstasy or mdma or ketamine or cocaine or hallucinogen* or inhalant*
S53	amphetamine* or methamphetamine*
S52	khat or qat
S51	alcohol* (drink* or consum*)
S50	(drug* or substance* or alcohol*) N1 (addict* or dependen* or problem* or abuse*)
S49	drug N1 (illicit* or illegal* or inject*)
S48	cannabis or marijuana
S47	heroin or opiate* or narcotic*
S46	MH Alcoholic Intoxication
S45	MH Drinking Behavior or MH Alcohol Drinking
S44	MH Alcoholic Beverages
S43	MH Alcohol-Related Disorders or MH Alcohol Abuse
S42	MH Substance Use Rehabilitation Programs or MH Alcohol Rehabilitation Programs or MH Drug Rehabilitation Programs
S41	MH Narcotics

S40	MH Amphetamine
S39	MH Substance Abusers
S38	SU Drug-Seeking Behavior
S37	MH Street Drugs
S36	MH Cannabis or MH Cocaine or MH Amphetamine
S35	MH Substance Abuse or MH Substance Dependence
S34	MH Substance Use Disorders
S33	S16 OR S17 OR S18 OR S19 OR S20 OR S21 OR S22 OR S23 OR S24 OR S25 OR S26 OR S27 OR S28 OR S29 OR S30 OR S31 OR S32
S32	dual diagnos*
S31	schizophreni* or<br>psychosis or psychoses or psychotic
S30	PTSD or posttrauma* or post?trauma* or post trauma*
S29	depress* or anxiet* or trauma*
S28	mental* N1 (health* or ill* or well* or disease* or disorder* or problem* or condition* or issue*)
S27	MH Psychotic Disorders or MH Schizophrenia or MH Affective Disorders, Psychotic or MH Organic Mental Disorders, Psychotic or MH Psychoses, Substance- Induced
S26	MH Injuries, Self-Inflicted
S25	MH Affective Disorders
S24	MH Phobic Disorders
S23	MH Trauma
S22	MH Suicide
S21	MH Stress Disorders, Post-Traumatic
S20	MH Anxiety or MH Anxiety Disorders
S19	MH Depression
S18	MH Mental Disorders
S17	MH Mental Health Services
S16	MH Mental Health

S15	S1 or S2 or S3 or S4 or S5 or S6 or S7 or S8 or S9 or S10 or S11 or S12 or S13 or S14
S14	displac* N1 (internal* or forced or mass or person* or people* or population*)
S13	(cultur* and linguistic* divers*) or (cultural* divers*) or CALD
S12	((ethnic* or racial) N1 (minor* or group* or communit* or background*))
S11	asylum N2 seek*
S10	migrant* or immigrant* or emigrant* or refugee*
S9	(non?english speak* or NESB or english as second language or english second language)
S8	MH Cultural Diversity or MH Minority Groups
S7	MH Acculturation
S6	MH Refugee Camps
S5	MH Refugees
S4	MH Immigrants
S3	MH Ethnic Groups
S2	SU transients and migrants
S1	(MM "Emigration and Immigration")
